# Effects of discrete dynamic-conductivity fractures on the transient pressure of a vertical well in a closed rectangular reservoir

**DOI:** 10.1038/s41598-017-15785-9

**Published:** 2017-11-14

**Authors:** Wanjing Luo, Pengcheng Liu, Qing Tian, Changfu Tang, Yinfang Zhou

**Affiliations:** 1 School of Energy Resources, China University of Geosciences, 29 Xueyuan Road, Beijing, 100083 China; 2 Exploration Research Institute, Anhui Provincial Bureau of Coal Geology, 20 Najing Road, Hefei, Anhui 230088 China; 3 School of Engineering, King’s College, University of Aberdeen, Fraser Noble Building, Aberdeen, AB24 3UE UK

## Abstract

With the extraction of hydrocarbons from reservoirs, fractures will gradually close and their permeabilities will be reduced. Therefore, fracture conductivity will change dynamically during hydrocarbon extraction. The main objective of this study is to develop a new semi-analytical model to simulate the flow inside a homogenous porous medium containing discretely distributed fractures with dynamic conductivities. Based on a dynamic-conductivity model, the pressure and pressure-derivative characteristics of a well on or near discrete dynamic-conductivity fractures were simulated. The results show that four flow regimes can be identified for a well near a dynamic-conductivity fracture. Dips in the pressure-derivative curves in the transitional flow period were observed as soon as pressure disturbances reached a fracture. In addition, humps caused by the effects of dynamic conductivity were observed after the transitional flow period. Wider and deeper dips were found on the pressure/pressure-derivative curves of a well surrounded by multiple fractures. The novel model presented here can provide a tool for elucidating the flow mechanisms of fluids in closed rectangular reservoirs with discretely dynamic-conductivity fractures.

## Introduction

Discrete fractures are fractures that are distributed in sandstone and carbonate formations and include unconnected natural fractures, faults and hydraulic fractures^1–3^. In many naturally fractured reservoirs, fractures and faults can be discrete rather than forming connected network systems^4,5^. The presence of discrete fractures contributes greatly to the enhanced production of oil and gas.

The fractures can be divided into three categories: uniform-flux fractures, infinite-conductivity fractures and finite-conductivity fractures. For uniform-flux fractures, we assume the flow rate along the fracture surface is uniform. The original uniform-flux/infinite-conductivity solutions for a vertically fractured well were developed by Gringarten *et al*.^6^. The wellbore pressure of an infinite-conductivity fracture can be obtained in the uniform-flux fracture case by measuring the pressure drop at 0.732 in the fracture. Cinco *et al*.^7^ found that the assumption of an infinite-conductivity fracture ignoring the pressure drop along the fracture may lead to errors for low and moderate fracture conductivities. The authors defined this type of low and moderate conductivity fracture as a finite-conductivity fracture.

Many studies have reported investigations of transient pressure analyses for vertical wells on single hydraulic fractures^6–11^ and horizontal wells intercepted by multiple hydraulic fractures^12–19^. Cinco and Samaniego^8^ discussed the flow regimes in detail for a finite-conductivity fracture. They first presented the entirety of flow regimes for a finite-conductivity fracture, i.e., bilinear flow, formation linear flow and radial flow regimes. Based on the flow regimes, reservoir parameters can be obtained through interpretations of well tests. Berumen *et al*.^9^ also investigated the pressure behaviours of wells intercepting asymmetric fractures of both infinite and finite conductivity under constant rates using numerical methods. Tiab *et al*.^10^ applied the Tiab’s Direct Synthesis technique to evaluate fracture asymmetry in finite-conductivity fracture wells producing at a constant rate. Luo and Tang^11^ proposed a model to discuss the pressure response of a varying-conductivity fracture. Larsen and Hegre^12^ rigorously presented a transient pressure solution for horizontal wells with circular finite-conductivity fractures in a three-dimensional unbounded formation using the Laplace transform method. Crosby *et al*.^13^ developed an analytical and experimental study of the initiation of transverse fractures from horizontal wells. Wan and Aziz^14^ described a new semi-analytical solution for horizontal wells with multiple hydraulic fractures. The fractures can be rotated at any horizontal angle to the well, and they need not fully penetrate the formation in the vertical direction. Wei and Economide^15^ provided a calculation procedure for transverse fractures and related the performance of each fracture with well-established methodologies such as the dimensionless productivity index. Medeiros *et al*.^16^ presented a discussion of diagnostic pressure and pressure-derivative plots for hydraulically fractured horizontal wells in locally and globally fractured formations. Brown *et al*.^17^ adopted a classic tri-linear flow model to simulate the pressure-transient and production behaviours of fractured horizontal wells in an unconventional shale reservoir. Zhao *et al*.^18^ presented a “tri-porosity” mathematical model to describe fluid flow from a shale gas formation to a multi-fractured horizontal well. Yuan *et al*.^19^ established a simple, practical and time-efficient septa-linear flow model to obtain the transient pressure and production.

Unlike hydraulic fractures that connect to the wellbore, discrete fractures and faults may not be connected to the wellbore but, rather, are at a distance. The effects of discrete fractures on pressure responses have also been studied^20–27^. Givens and Crawford^20^ studied the influence of isolated fractures on fluid-displacement response. They reported that the fracture orientation and length and the fracture-to-well distance are the key parameters that control well performance. Based on assumptions of steady-state-flow behaviour and uniform fracture distribution, Huskey and Crawford^21^ studied the effect of isolated vertical fractures on pressure distribution. Cinco *et al*.^22^ developed an analytical model to study the transient-flow behaviours of a well near a single natural infinite-conductivity fracture in an infinite reservoir. Guo and Evans^23^ presented the pressure-transient behaviours of a horizontal well penetrated by multiple randomly distributed vertical fractures in an infinite reservoir and bounded reservoir. Izadi and Yildiz^24^ presented a semi-analytical model for transient flows into multiple vertical wells producing from a porous medium containing randomly distributed discrete fractures. Zeng *et al*.^25^ discussed the pressure response for a vertical well with discrete fractures by accounting for the effects of non-Darcy flow. Based on their work, three flow regions, i.e., fluid flow near the wellbore, fracture-dominated fluid flow and fluid flow in the matrix away from the fracture can be identified. Biryukov and Kuchuk^26,27^ presented a mesh-free semi-analytical solution for pressure-transient behaviour in a 2 Dimensional infinite reservoir containing a network of discrete or connected finite- and infinite-conductivity fractures.

The fracture conductivities reported in the above literature were assumed to be constant. However, a reduction in formation pressure will result in increases in effective closure stress on the discrete fractures in an oilfield. Hence, fractures will gradually close, leading to dynamic decreases in fracture permeability and conductivity with time. A transient pressure analysis for a vertical well on a dynamic-conductivity fracture has been investigated using semi-analytical and numerical methods^28–33^. Chen and Evers^28^ developed a simple model to illustrate the performance of a fractured well with stress-sensitive conductivity. Berumen and Tiab^29^ revealed the effects of pressure on permeability and conductivity using a numerical method. Pedroso and Correa^30^ developed a new model to discuss the effects of permeability on the flow behaviour of a fractured well. Cho *et al*.^31^ presented experimental data for a pressure-dependent natural facture. Zhang *et al*.^32^ presented the results of a study that analysed the build-up of pressure in a vertically fractured well while considering stress-sensitive permeability and hysteresis effects in fractures. Wang and Aryana^33^ investigated how stress-dependent fracture apertures and their spatial variations affected production from unconventional gas reservoirs with complex fracture geometries.

As noted above, the transient pressure behaviours of wells due to constant-conductivity fractures^6–19^ and dynamic-conductivity fractures have been discussed^28–33^. In addition, transient pressure responses of wells near constant-conductivity fractures have been studied^20–27^. However, to the best of our knowledge, few studies have discussed the transient pressure behaviours of wells near discrete fractures with dynamic conductivities in closed rectangular reservoirs. In this paper, we develop a new semi-analytical method that is an extension of the popular transient pressure calculation method proposed by Cinco *et al*.^7,8^ to examine the pressure responses of a vertical well in a reservoir with discrete dynamic-conductivity fractures. The oil or gas is assumed to be produced only through the vertical well.

This paper presents a novel semi-analytical model of a vertical well with dynamic-conductivity fractures in a rectangular reservoir. Two verification cases (a well on a dynamic-conductivity fracture in an infinite reservoir and a well near a constant-conductivity fracture in an infinite reservoir) and the effects of various parameters on the dimensionless pressure of a vertical well are studied. The effects of boundary size and dynamic conductivity on the dimensionless pressure of a vertical well on a fracture are presented. Additionally, the effects of fracture location, initial conductivity, dynamic conductivity, and distance between the well and fracture on the dimensionless pressure of a vertical well in the vicinity of discrete fractures are presented. Finally, the effects of fracture number and dynamic conductivity on the dimensionless pressure of a vertical well around four fractures are presented.

## Mathematical Models

### Basic assumptions

**Figure 1:**
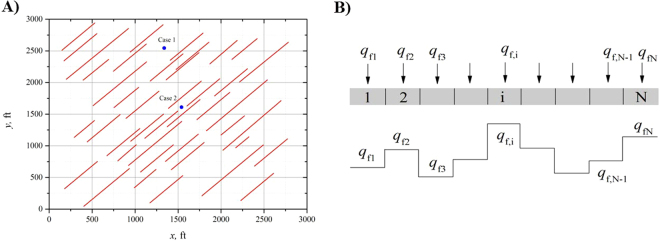
Schematic of the physical model in a closed rectangular reservoir. (**A**) Well with discrete fractures. (**B**) Uniform-flux discretization model.

In this paper, we assume that the discrete fractures are not joined and that the oil or gas is produced only through a vertical well (Fig. 1A). Additional basic assumptions are given below.

1. The closed rectangular reservoir is isotropic and homogeneous. The reservoir has constant thickness *h*, constant porosity *φ* and constant permeability *k*. The reservoir contains a slightly compressible single-phase fluid of constant compressibility *c*_t_ and constant viscosity *μ*.
2. The reservoir is fully penetrated by vertically discrete fractures. No fluids are assumed to flow into the fractures at their tips. Moreover, the fluids in the fractures are assumed to be incompressible. Each fracture is divided into multiple segments of equal length and uniform flux (Fig. 1B).
3. Production of a vertical well with a constant rate from a closed reservoir leads to a reduction in fluid pressure and subsequent increase in net closure stress on the fractures. Fracture permeability changes with formation pressure. Thus, the dynamic conductivity of a discrete fracture is used to describe this phenomenon. The flows in the formation and fractures are assumed to obey Darcy’s law.

### Model of fracture dynamic conductivity

According to the effective stress law, any change in effective stress must be compensated for changes in pore pressure, indicating that the variation of permeability caused by a stress change can be expressed as a function of pore pressure. Experiments and exponential models have been used to characterize the relationship between pressure and permeability^28–33^.

We chose a more general model used by Zhang *et al*.^32^ for this paper. The relationship between fracture permeability and pore pressure is described as

1 $$\frac{{k}_{f}({p}_{f})-{k}_{f\min }}{{k}_{f}({p}_{i})-{k}_{f\min }}={e}^{-{\gamma }_{f}({p}_{fi}-{p}_{f})}$$

The fracture permeability can be written as

2 $${k}_{f}({p}_{f})={k}_{f\min }+({k}_{f}({p}_{i})-{k}_{f\min })\cdot {e}^{-{\gamma }_{f}({p}_{fi}-{p}_{f})}$$

where: Subscripts “*f*” is fracture property; “*min*” is minimum;“*i*” is initial or segment; *k*_*f*_ is fracture permeability, mD; p is pressure, psi; *γ*_*f*_ is fracture permeability modulus.

According to the definitions of the dimensionless parameters (Appendix A), the dimensionless dynamic conductivity of a fracture is

3 $${C}_{fD}({p}_{fD})={C}_{fDi}\cdot [(1-\frac{{C}_{f{D}{\rm{\min }}}}{{C}_{fDi}})\cdot \exp (-{\gamma }_{fD}\cdot {p}_{fD})+\frac{{C}_{f{D}{\rm{\min }}}}{{C}_{fDi}}]$$

Note that *C*_*fD*_ is not constant but, rather, a function of the dimensionless fracture pressure ${p}_{fD}$. In other words, *C*_*fD*_ evolves spatially and temporally, and Eq. 3 can be further expressed as

4 $$\frac{{C}_{fD}}{{C}_{fDi}}=(1-\frac{{C}_{f{D}{\rm{\min }}}}{{C}_{fDi}})\cdot \exp (-{\gamma }_{fD}\cdot {p}_{fD})+\frac{{C}_{f{D}{\rm{\min }}}}{{C}_{fDi}}$$

where: Subscripts “*D” is* dimensionless; *C*_*fD*_ is the initial fracture conductivity corresponding to the initial formation pressure.

### Model of fluid flow in the reservoir

The wing model introduces a way to flexibly generate complex fractures when describing discrete fractures^34^. We assume that there are *M* wings and a vertical well in a closed rectangular reservoir. The well can be on a fracture or near fractures. The flow rate in the wellbore is *q*_w_. The *m*-th wing is divided equally into *N*_m_ segments with uniform fluxes (Fig. 1B). The dimensionless pressure of each segment can be obtained in the Laplace domain using the superposition principle.

(1) The well is on the fracture.

5 $${\bar{p}}_{Dij}=\sum _{k=1}^{M}\sum _{m=1}^{{N}_{k}}{\bar{q}}_{Dkm}\cdot s{\bar{p}}_{ufD(ij,km)}({x}_{wDij},{y}_{wDij},{x}_{wDkm},{y}_{wDkm},{x}_{eD},{y}_{eD},{L}_{fDkm})$$

(2) The well is not on the fracture.

For each segment,

6 $$\begin{array}{c}{\bar{p}}_{Dij}=s{\bar{q}}_{wD}{\bar{p}}_{uwDij}({x}_{wDij},{y}_{wDij},{x}_{wD},{y}_{wD},{x}_{eD},{y}_{eD})\\ \quad \quad \,\,\,+\sum _{k=1}^{M}\sum _{m=1}^{{N}_{k}}{\bar{q}}_{Dkm}\cdot s{\bar{p}}_{ufD(ij,km)}({x}_{wDij},{y}_{wDij},{x}_{wDkm},{y}_{wDkm},{x}_{eD},{y}_{eD},{L}_{fDkm})\end{array}$$

For the well,

7 $$\begin{array}{c}{\bar{p}}_{wD}={\bar{q}}_{wD}\cdot s{\bar{p}}_{uwD}({x}_{wD},{y}_{wD},{x}_{wD},{y}_{wD},{x}_{eD},{y}_{eD})\\ \quad \quad \,\,\,+\sum _{k=1}^{M}\sum _{m=1}^{{N}_{k}}{\bar{q}}_{Dkm}\cdot s{\bar{p}}_{ufD(km)}({x}_{wD},{y}_{wD},{x}_{wDkm},{y}_{wDkm},{x}_{eD},{y}_{eD},{L}_{fDkm})\end{array}$$

where: the subscript “*w”* is wellbore property; “*ij*” indicates the *j-th* segment of the *i-th* fracture wing; “*e*” is boundary; *M* is the numbers of wings; *N*_k_ is segments with uniform fluxes; ${\bar{p}}_{ufD(ij,km)}$ and ${\bar{p}}_{uwDij}$ are the changes in dimensionless pressure at the *j-th* segment of the *i-th* wing caused by the production of the *m-*th segment of the *k-*th wing and the vertical well in the Laplace domain, respectively; *x*_*D*_ and *y*_D_ are dimensionless coordinate in the *x* and *y* direction, respectively; *x*_eD_ and *y*_eD_ are dimensionless boundary coordinate in the *x* and *y* direction, respectively.

The uniform-flux fracture solution ${\bar{p}}_{ufD(ij,km)}$ and point source solution ${\bar{p}}_{uwD}$ in a rectangular reservoir can be obtained from the literature^35,36^.

### Model of fluid flow in the fracture

For fluid flow in a dynamic-conductivity fracture, the fracture conductivity *C*_*fD*_ is correlated with ${p}_{fD}$. Because ${p}_{fD}$ is changing spatially and temporally, the fracture can be considered to exhibit varying conductivity at each step.

**Figure 2:**
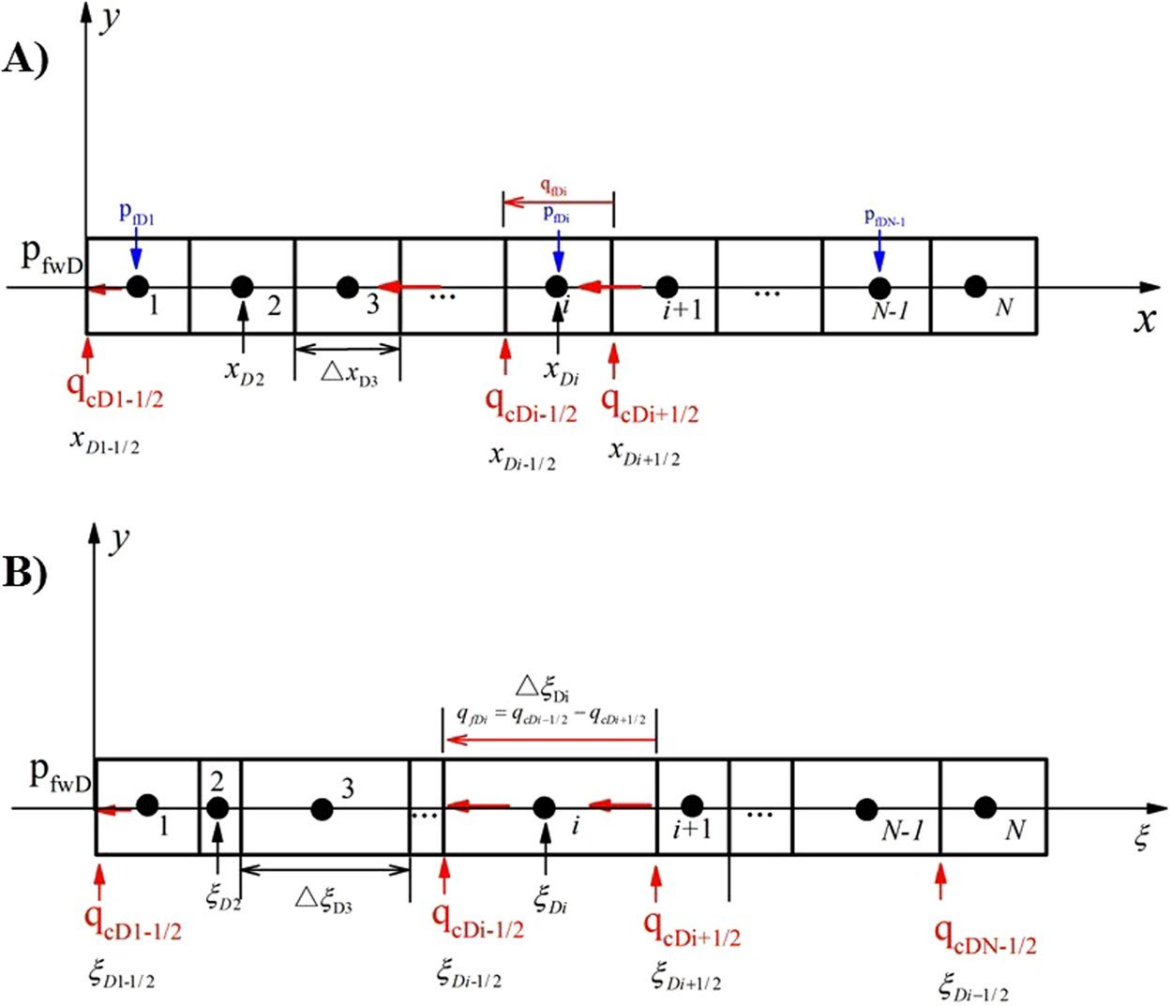
Schematic of a dimension transformation. (**A**) A varying-conductivity, equal-length fracture. (**B**) A constant-conductivity, unequal-length fracture.

A dimension transformation method is introduced to convert the varying-conductivity, equal-length fracture into a constant-conductivity, unequal-length fracture (Appendix B, Fig. 2)^11^.

A detailed derivation of fluid flow in a dynamic-conductivity fracture is presented in Appendix B. After the transformation, the equation for a varying-conductivity fracture at each time step can be written as

8 $${\bar{p}}_{fwD}-{\bar{p}}_{fDi}=(\frac{2\pi }{{C}_{fD}})\cdot [\begin{array}{c}{\xi }_{Di}\cdot \sum _{j=1}^{N}{\bar{q}}_{fDj}-\frac{{({\xi }_{Di}-{\xi }_{Di-1/2})}^{2}}{2\cdot {\rm{\Delta }}{y}_{Di}}\cdot {\bar{q}}_{fDi}\\ -\sum _{j=1}^{i-1}(\frac{{\rm{\Delta }}{\xi }_{Dj}}{2}+{\xi }_{Di}-\sum _{n=1}^{j}{\rm{\Delta }}{\xi }_{Dn})\cdot {\bar{q}}_{fDj}\end{array}]$$

where: *ξ*_*D*_ is dimensionless coordinate in the *ξ* direction; ${\rm{\Delta }}{\xi }_{Di}$ and ${\rm{\Delta }}{\xi }_{Dn}$ are dimensionless discretized step of the *i-th* and *n-th* segments in the *ξ* direction; ${\rm{\Delta }}{y}_{Di}$dimensionless discretized step of the *i-th* segment in the *y* direction

### Coupling model

According to the continuity condition whereby pressure and flux must be continuous along a fracture’s surface, the following conditions must hold along the fracture plane:

9 $${\bar{p}}_{fD}={\bar{p}}_{D},{\bar{q}}_{fD}={\bar{q}}_{D}.$$

If the well is on the fracture,

10a $$\sum _{j=1}^{Ni}{\bar{q}}_{fDij}=1/s,\,{q}_{wD}=0,\,\sum _{j=1}^{{N}_{k}}{\bar{q}}_{fDkj}=0,k=1\cdot \cdot \cdot i-1,i+1,\cdot \cdot \cdot M.$$

If the well is not on the fracture,

10b $${\bar{q}}_{wD}=1/s,\,\sum _{j=1}^{{N}_{k}}{\bar{q}}_{fDkj}=0,k=1\cdot \cdot \cdot M.$$

where: *s* is time variable in Laplace domain, dimensionless; ${\bar{q}}_{wD}$ dimensionless wellbore flow rate of a well, in Laplace domain, dimensionless; ${\bar{q}}_{fDkj}$ is dimensionless wellbore flow rate of a fracture at “*kj*” in Laplace domain, dimensionless.

For the simultaneous calculation of Eqs (4–10), the pressure solution can be obtained via the Gaussian elimination method and can further be inverted to the time domain using the Stehfest numerical algorithm. With production, fracture pressure will gradually decrease, and the dynamic conductivity should be updated along the fracture at each time step. Based on the semi-analytical solution, an iterative program can be used to calculate the dimensionless pressure and flux distribution at each time step (Appendix C). The conductivity distributions along the fracture can then be obtained for each time step.

## Model verification

To the best of our knowledge, there are no existing models for discrete fractures of dynamic conductivity for comparison. In this section, two specific models, i.e., a well near a discrete fracture of constant conductivity and a well on a fracture with dynamic conductivity, are used to verify our model.

### Comparison with a well near a discrete fracture with constant conductivity

**Figure 3:**
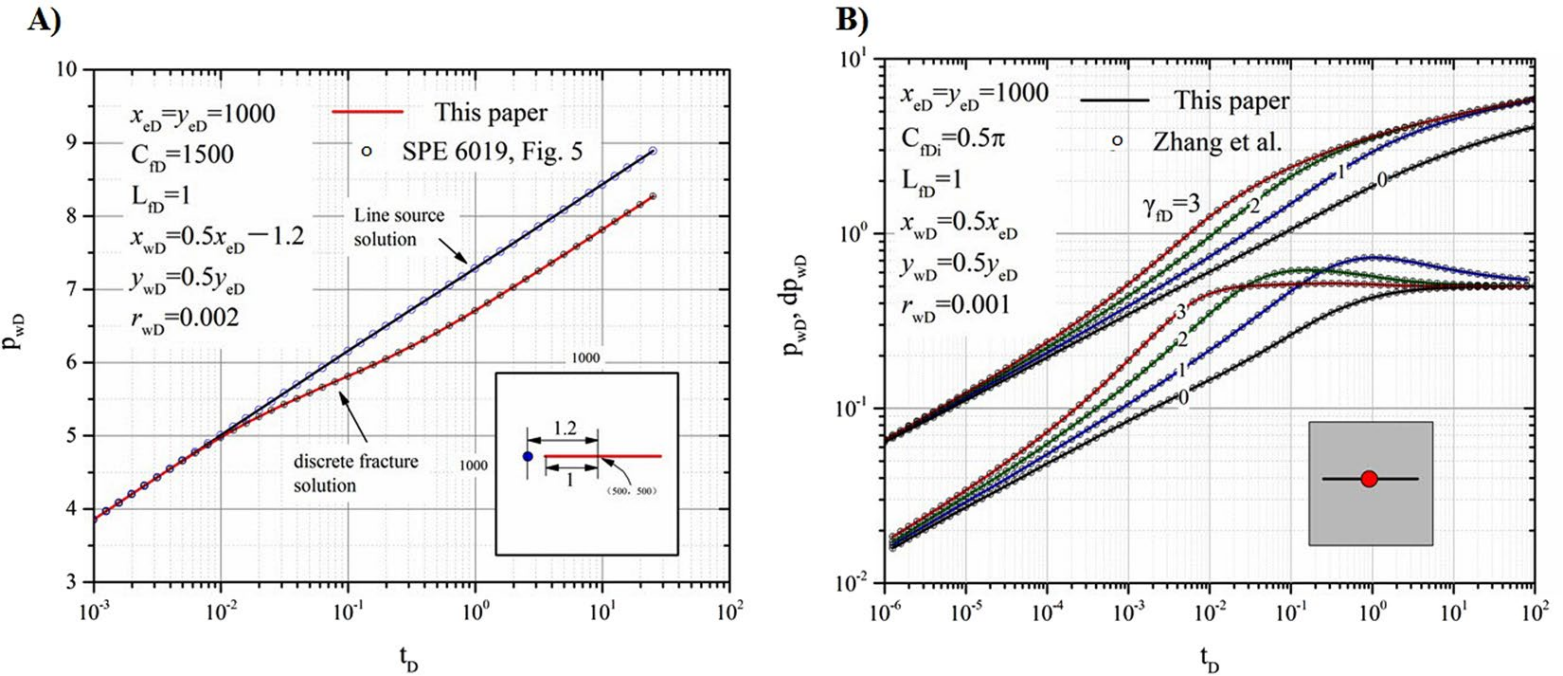
Comparison of our model with other models. (**A**) Cinco-Ley *et al*. model. (**B**) Zhang *et al*. model.

A transient-flow solution for an infinite-conductivity discrete fracture near a vertical well was presented by Cinco *et al*. in a real domain^22^. The calculation parameters are listed in Fig. 3(A).

### Comparison with a fractured well with varying conductivity

Zhang *et al*.^32^ developed a finite-difference numerical simulator to model a vertical well on a symmetric fracture of varying conductivity in an infinite reservoir. Figure 3(B) displays the comparison of our model with other models for a constant fracture conductivity.

As shown in Fig. 3(A), our model is in significant agreement with the model of Cinco *et al*.^22^. (the solid lines are the results from our model, and the circles with crosses are from the model of Cinco *et al*.^22^). As shown in Fig. 3(B), our semi-analytical model (solid lines) agrees closely with the numerical solution of the Zhang *et al*. model^32^. The circles represent a well on a symmetric fracture with an initial conductivity = 0.5π. The upper group of curves is dimensionless pressure, and the lower group is its derivative. The red, green, blue and black lines represent the values for *γ*_*fD*_ = 3, 2, 1 and 0, respectively. If *γ*_*fD*_ = 0, the fracture conductivity remains constant for all time, and if *γ*_*fD*_ > 0, the conductivity changes with production.

The model verification proves that our model is accurate and that it can be further used in calculations for complex cases.

## Results and Discussions

We focus on the effects of dimensionless permeability modulus *γ*_*fD*_, discrete-fracture-well distance D, initial discrete-fracture conductivity *C*_*fDi*_ and boundary size ${x}_{eD}\times {y}_{eD}$ on dimensionless pressure. Based on the new semi-analytical method presented above, three fundamental cases are presented in detail.

**Figure 4:**
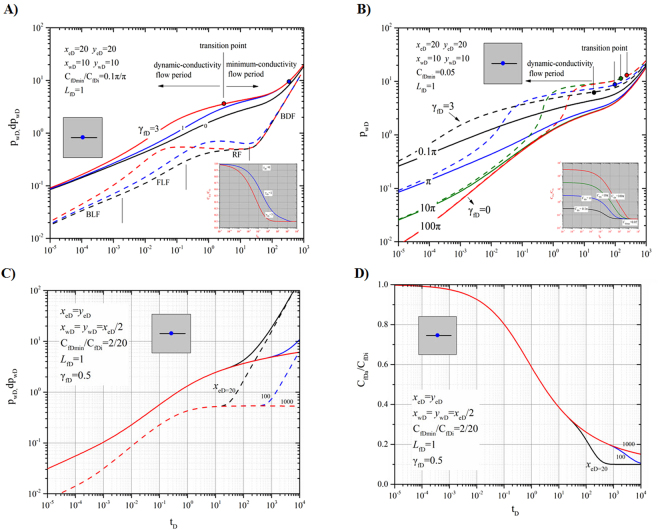
Effects of fracture permeability modulus and initial dimensionless fracture conductivity on pressure. (**A**) Dimensionless pressure (solid lines) and its derivative (dash lines) of a well on a symmetric fracture with different dimensionless fracture permeability moduli. (**B**) Dimensionless pressure of a well on a symmetric fracture with different initial dimensionless fracture conductivities. (**C**) Effects of boundary size on dimensionless pressure and its derivative on a symmetric fracture. (**D**) The ratio of average conductivity to initial conductivity over time for different boundary sizes.

Case 1 is a pressure-transient analysis of a vertical well on a fracture, where the effects of boundary size, dynamic conductivity and dimensionless permeability modulus on the dimensionless pressure and pressure derivative are addressed (Fig. 4).

**Figure 5:**
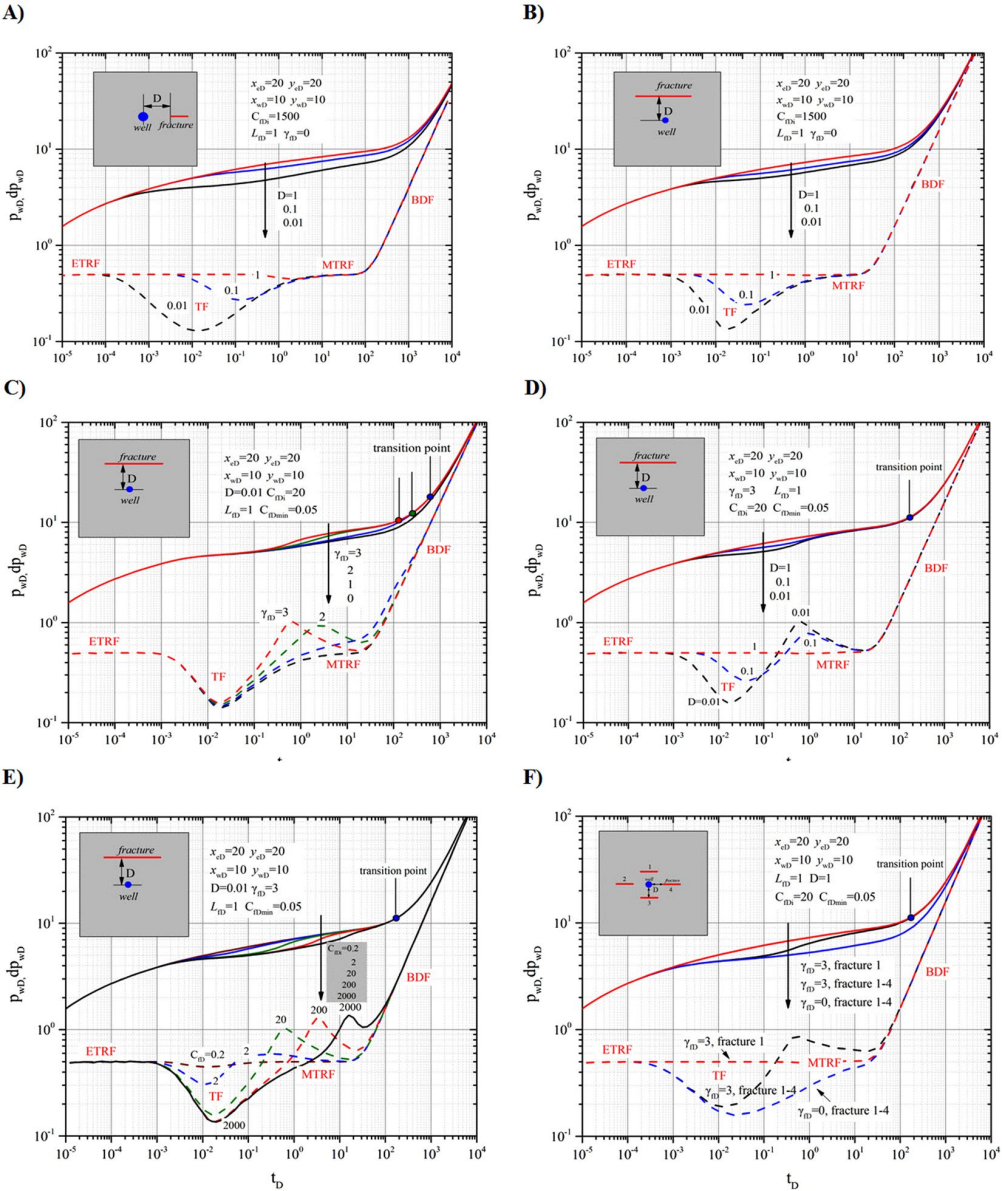
Effects of parameters on pressure response. (**A**) Effects of dimensionless distance on dimensionless pressure and derivative for collinear well and fracture. (**B**) Effects of dimensionless distance on dimensionless pressure and derivative for well perpendicular to fracture. (**C**) Effects of dimensionless fracture permeability modulus on dimensionless pressure and its derivative. (**D**) Effects of dimensionless distance on dimensionless pressure and derivative. (**E**) Effects of dimensionless initial fracture conductivity on dimensionless pressure and its derivative. (**F**) Comparison of dimensionless pressure and its derivative for a well near a discrete fracture and one surrounded by multiple fractures.

Case 2 is a pressure-transient analysis of a vertical well in the vicinity of discrete fractures. This case focuses on the effects of fracture conductivity, dimensionless permeability modulus, fracture number, distance between the well and fracture on the dimensionless pressure and pressure derivative (Fig. 5).

**Figure 6:**
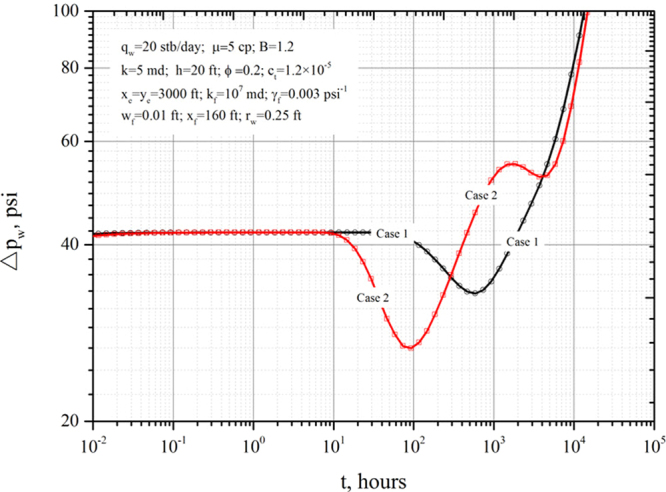
Pressure derivative in a vertical open hole producing from a formation containing discrete fractures.

Case 3 is a case study employing synthetic data to obtain the pressure derivative for the discrete-fracture-well system (Fig. 6).

### Pressure-transient analysis of a vertical well on a fracture

A fracture is assumed to be located at the centre of a closed square reservoir with ${x}_{eD}={y}_{eD}=20$. A vertical well on the fracture is under production at a constant production rate.

#### Effects of dimensionless permeability modulus

Figure 4 presents the curves of dimensionless pressure (solid line) and pressure derivative (dashed line) vs. dimensionless time for a symmetrical fracture with different dimensionless permeability moduli *γ*_*fD*_. In Fig. 4(A), the red, blue and black lines represent the values for *γ*_*fD*_ = 3, 1 and 0, respectively. For *γ*_*fD*_ = 0 (black lines), i.e., constant fracture conductivity, the characteristic flow regimes can be identified on the derivative curve: bilinear flow with a slope of 1/4, formation linear flow with a slope of 1/2, radial flow with a constant value of 1/2 and boundary-dominated flow with a slope of 1. The sub-figure presents the ratios of the average fracture conductivities *C*_*fDa*_ to initial fracture conductivities *C*_*fDi*_ over time for different dimensionless fracture permeability moduli *γ*_*fD*_: 3(red line), 1 (blue line) and 0 (horizontal black line). The transition point is defined as the time corresponding to the occurrence of the minimum fracture conductivity ${C}_{fD\min }$. For *γ*_*fD*_ = 0 (black lines), no transition point exists. For *γ*_*fD*_ = 3 (red line) and *γ*_*fD*_ = 1 (blue line), the transition points can be found (marked by the red solid and blue solid circles, respectively) on the dimensionless pressure curves. Before the transition points, the fracture makes a dynamic contribution to well production. We define the time before a transition point as the dynamic-conductivity flow period. After that time, the fracture conductivity will remain constant and at a minimum ${C}_{fD\min }$.

We set the initial *C*_*fDi*_ and minimum fracture conductivities ${C}_{fD\min }$ to π and 0.1π, respectively. As shown in Fig. 4(A) (sub-figure), an obvious decrease in dimensionless fracture conductivity *C*_*fD*_ can be observed with decreasing dimensionless permeability modulus *γ*_*fD*_.With increasing dimensionless permeability modulus *γ*_*fD*_, dimensionless pressure increases, which means that a larger pressure drop is needed to maintain a constant production rate. In addition, due to the influence of permeability modulus (*γ*_*fD*_ > 0), the pressure and pressure-derivative curves deviate from the type match curve (*γ*_*fD*_ = 0). Thus, the characteristic flow regime for a symmetric fracture (bilinear flow with a slope of 1/4, formation linear flow with slope of 1/2 and radial flow with the derivate of 1/2) cannot be identified.

We define the intervals when the flow exhibits dynamic fracture conductivity and minimum fracture conductivity as dynamic-conductivity flow and minimum-conductivity flow periods, respectively. For small permeability moduli *γ*_*fD*_, long dynamic flow periods and high oil recoveries can be expected. The transition points between two flow periods for *γ*_*fD*_ = 1 and *γ*_*fD*_ = 3 are marked with a solid circle in Fig. 4(A). A rapid decrease in dynamic fracture conductivity *C*_*fD*_ with high permeability modulus *γ*_*fD*_ (Fig. 4(A) sub-figure) results in a small dimensionless time corresponding to the transition point such as *t*_D_ = 3 for *γ*_*fD*_ = 3 and *t*_D_ = 300 for *γ*_*fD*_ = 1, which indicates that fracture effectiveness decreases with increasing values of *γ*_*fD*_.

#### Effects of dimensionless initial fracture conductivity

Figure 4(B) illustrates the effect of initial fracture conductivity *C*_*fDi*_ on dimensionless pressure with (dash line, *γ*_*fD*_ = 3) and without (solid line, *γ*_*fD*_ = 0) considering the effects of the permeability modulus. In Fig. 4(B), the red, green, blue and black lines correspond to the values of initial fracture conductivity, *C*_*fDi*_ = 100π, 10π, π and 0.1π. The ratios of the average fracture conductivities *C*_*fDa*_ to initial fracture conductivities *C*_*fDi*_ for different initial dimensionless fracture conductivities *C*_*fDi*_ are illustrated in the sub-figure of Fig. 4(B). Four transition points are identified on the dimensionless pressure curves (dashed lines) for *γ*_*fD*_ = 3.

For the purpose of comparison, we specified that the minimum fracture conductivities were all ${C}_{fD\min }$ = 0.05. For low initial conductivities, for example, *C*_*fDi*_ = 0.1π, the effect of permeability modulus v on dimensionless pressure is relatively stable (no apparent climbing) throughout the entire production period. As initial fracture conductivity increases, the effect of permeability modulus *γ*_*fD*_ on dimensionless pressure will be delayed. For *C*_*fDi*_ = 100π, the pressure responses for *γ*_*fD*_ = 0 and *γ*_*fD*_ = 3 are nearly indistinguishable for *t*_D_ less than 1. However, a stronger effect gradually appears for larger dimensionless times, such that *t*_D_ = 10^−2^–10^−1^ for *C*_*fDi*_ = π, 10^−1^–10^0^ for *C*_*fDi*_ = 10π and 10^0^–10^1^ for *C*_*fDi*_ = 100π, where significant increases in dimensionless pressure can be observed. In addition, longer dynamic-conductivity flow periods resulting from larger initial conductivities can be observed from the curves, where the transition points are marked as solid circles. Therefore, large initial fracture conductivities *C*_*fDi*_ can delay and strengthen the effects of permeability modulus *γ*_*fD*_ on dimensionless pressure. As shown in the sub-figure of Fig. 4(B), it takes longer for *C*_*fD*_ to decrease to the minimum conductivity ${C}_{fD\min }$ as *C*_*fDi*_ increases.

#### Effects of boundary size

Figure 4(C,D) demonstrates the effects of boundary size on pressure response at *γ*_*fD*_ = 0.5 and *C*_*fDi*_ = 20. Figure 4(C) displays the effects of boundary size on dimensionless pressure and its derivative for a well on a symmetric fracture at *γ*_*fD*_ = 0.5. The red, blue and black lines correspond to the boundary size values: ${x}_{eD}={y}_{eD}$ = 1000, 100 and 20. The dimensionless pressures and their derivatives are represented by the solid and dashed lines, respectively. Figure 4(D) displays the ratios of average conductivity *C*_*fDa*_ to initial conductivity *C*_*fDi*_ over time for different boundary sizes at *γ*_*fD*_ = 0.5. The red, blue and black lines correspond to the boundary size values, ${x}_{eD}={y}_{eD}$ = 1000, 100 and 20, respectively.

Figures 4(C) and (D) show that the small distance between the well and boundary not only caused the boundary-dominated flow (BDF) to occur earlier but also accelerated the declining rate of the average conductivity of the fracture. Apparently, the existing boundary will significantly impact fracture conductivity and produce notable differences when the pressure response reaches it. This phenomenon is caused by the rapid drop in the average pressure after the boundary-dominated-flow period, which further leads to obvious decreases in dynamic fracture conductivity *C*_*fD*_. Thus, a shorter distance to the boundary will enhance the effect of permeability modulus *γ*_*fD*_ on dimensionless pressure.

### Pressure-transient analysis of a vertical well in the vicinity of discrete fractures

For discretely fractured reservoirs, a well may be drilled in the vicinity of discrete fractures with dynamic conductivities. The production of the well will be affected by the fractures. In this section, we will discuss the pressure behaviours of a vertical well in the vicinity of discrete fractures within a closed square reservoir with ${x}_{eD}={y}_{eD}$ = 20.

#### Effect of the constant fracture conductivity on the dimensionless pressure

To reveal the influence of the relationship between the well and a constant-conductivity fracture (*C*_*fD*_ = *C*_*fDi*_ = 1500, *γ*_*fD*_ = 0) on dimensionless pressure, we set the dimensionless distance to 0.01, 0.1, and 1.0.

Figure 5(A,B) demonstrates the effects of dimensionless distance between the well and discrete fracture on dimensionless pressure and its derivative (the well and fracture are collinear) (*γ*_*fD*_ = 0). Four flow regimes on the derivative curve are shown (black dashed line), i.e., early-time radial flow, transitional flow, middle-time pseudo-radial flow, and boundary-dominated flow. Dimensionless pressure and its derivative are represented by solid and dashed lines, respectively. The red, blue and black lines correspond to dimensionless distances of 1, 0.1 and 0.01, respectively. Figure 5(A) shows that the well and fracture are collinear. Figure 5(B) shows that the well is perpendicular to the fracture.

The durations of the transitional flows for Fig. 5(A) and (B) differed, as shown in the figures. After a period of middle-time pseudo-radial flow, a propagating pressure front would approach the fracture and transitional flow would occur. In that flow period, downward dips in the derivative curves can be observed. The durations and depths of the dips depend on the well-fracture distance. With increasing dimensionless distance, dips become narrower and shallower, representing the gradually decreasing effect of the fracture on wellbore pressure. When the distance is greater than 1, transitional flow is not detected. A comparison of Fig. 5(A) and (B) shows the stronger influence of the collinear case (wider and deeper dip, Fig. 5(A)) than that of the perpendicular case (Fig. 5(B)).

After the transitional flow period, middle-time pseudo-radial flow with a pressure derivative of 0.5 occurs. Finally, boundary-dominated flow is achieved. In the middle-time pseudo-radial flow and boundary-dominated flow period, the curves of pressure-derivative overlap.

#### Effects of dynamic fracture conductivity on dimensionless pressure

We now focus on the effects of permeability modulus *γ*_*fD*_, dimensionless distance and initial fracture conductivity *C*_*fDi*_ on dimensionless pressure with a dynamic-conductivity fracture near a vertical well. The parameters used for the calculations are listed on the figures.

1. **Effects of permeability modulus**. Figure 5(C) shows the effects of permeability modulus *γ*_*fD*_ on dimensionless pressure. Dimensionless pressure and its derivative are represented by solid and dashed lines, respectively. The red, green, blue and black lines correspond to the dimensionless permeability modulus values,*γ*_*fD*_ = 3, 2, 1, and 0, respectively. The effect of *γ*_*fD*_ on dimensionless pressure derivative is concentrated in the middle-time pseudo-radial flow period. Three transition points can be seen. The presence of the dynamic fracture conductivity causes the pressure-derivative curves to deviate from the constant-conductivity curve (*γ*_*fD*_ = 0). A hump can be found in the middle-time pseudo-radial flow and boundary-dominated flow periods, which indicates the occurrence of sharp pressure drops, and accordingly, the middle-time pseudo-radial flow is invisible.
2. **Effects of distance**. Figure 5(D) presents the effects of dimensionless distance on the pressure responses. Dimensionless pressure and its derivative are represented by solid lines and dashed lines, respectively. The red, blue and black lines correspond to dimensionless distance values of 1, 0.1 and 0.01, respectively. A hump caused by the effects of dynamic conductivity in the middle-time pseudo-radial flow period can be found in the pressure-derivative curves by comparing Fig. 5(D) (*γ*_*fD*_ = 3) with Fig. 5(B) (*γ*_*fD*_ = 0). Note that when the dimensionless distance is greater than 1, the effect of the fracture can be ignored.
3. **Effects of initial fracture conductivity**. Figure 5(E) demonstrates the effects of initial fracture conductivity *C*_*fDi*_ on dimensionless pressure and its derivative with dynamic fracture conductivity (*γ*_*fD*_ = 3). Dimensionless pressure and its derivative are represented by solid and dashed lines, respectively. The wine, blue, green, red and black lines correspond to the initial conductivity values (*C*_*fDi*_ = 0.2, 2, 20, 200 and 2000, respectively). As shown in Fig. 5(E), the dip in the transitional flow period deepens and the height of the hump on the pressure-derivative curve in the middle-time pseudo-radial flow period increases with increasing initial fracture conductivity. For extremely small initial fracture conductivities such as *C*_*fDi*_ = 0.2, the dips and humps nearly disappear.

Based on the discussion of a dynamic-conductivity fracture near a well, the presence of fractures benefits production, which leads to reductions in pressure drop (dips). Wider and deeper dips can be obtained when fractures are close to wells. In contrast, due to the decrease in dynamic conductivity with production, a hump can be observed in the middle-time pseudo-radial flow period, which indicates that a steep increase in pressure drop occurred. Thus, it is very important to make use of the early-time radial flow and transitional flow periods for production. For the middle-time pseudo-radial flow and boundary-dominated flow periods, the fracture negatively impacts production because the sharp declines in dynamic conductivity result in large pressure drops (humps).

### Pressure-transient analysis of a vertical well near multiple fractures

To illustrate the effects of fracture number, we set 4 fractures around a vertical well. The dimensionless pressures and their derivatives for a well near a discrete fracture or multiple fractures at dimensionless distance *D* = 1 are compared in Fig. 5(F). In Fig. 5(F), dimensionless pressure and its derivative are represented by solid lines and dashed lines, respectively. The red lines correspond to a well near a fracture with *γ*_*fD*_ = 3. The black lines correspond to a well surrounded by 4 fractures with *γ*_*fD*_ = 3. The blue lines correspond to a well surrounded by 4 fractures with *γ*_*fD*_ = 0.

From Fig. 5(F), when compared against the case with a fracture near a well (red line), an obvious dip can be found on the pressure-derivative curve for four fractures around a well (blue line). Therefore, to maintain a consistent outcome, lesser pressure drops are needed for a well surrounded by multiple fractures. Similar to the single fracture near a well, a hump on the pressure-derivative curve is found after transitional flow (black line).

### Case study

Here, a case employing synthetic data is used to examine the pressure-derivative characteristics of a vertical open hole producing from a formation dissected by 42 discrete fractures with lengths between 100 and 450 ft (Fig. 1). All the fractures deviate from the horizontal axis by an azimuthal angle of 50 degrees. The fracture lengths and locations were randomly selected. Figure 1 shows the fracture pattern and distribution in a Cartesian coordinate system. Only one vertical open hole is producing from the discrete fractured formation at a constant production rate (*q*_w_ = 20 stb/day). The well location in the formation is variable.

We consider two different cases for the well location. The positions of the vertical open hole in each case are marked with solid circles in Fig. 1. In Case 1, the vertical open hole is located at *x*_w_ = 1335 ft and *y*_w_ = 2533 ft. In this case, the vertical well is relatively distant from the nearby fractures and close to the boundary. In Case 2, the well is positioned near the centre of the reservoir at *x*_w_ = 1520 ft and *y*_w_ = 620 ft and is very close to four discrete fractures. The basic data for the reservoir and fluid used in the simulations are listed in Fig. 6. Figure 6 presents the pressure-derivative responses of a vertical well in a closed rectangular reservoir with discrete fractures. Figure 6 indicates that the expected three flow regimes—early-time radial flow, transitional flow period and boundary-dominated flow—are observed in both cases.

At very early times, only the formation around the vertical well contributes to flow. The pressure disturbance created at the wellbore has not yet arrived at the fracture. The logarithmic pressure derivative during the early-time radial flow period is constant at 40.8 psi. At this time, there is no fluid flow in or across the fracture. As soon as the pressure disturbances generated at the wellbore reaches the fracture, flows in and around the fracture are triggered and transitional flow occurs. During the transitional flow period, the pressure derivative decreases and then increases. Obvious dips can be observed on the curves. Comparing Case 1 with Case 2, a deeper dip is seen for Case 2 due to the effect of multiple fractures around the wellbore. The duration of the transitional flow period is controlled by fracture length, distance to fracture, fracture prosperities and fracture number.

After the transitional flow period, given the same discrete-fracture field, the location of the vertical open hole makes a significant difference in the derivative response during the late-time flow period. The position of the wellbore in Case 1 is close to the boundary and away from the fractures, the boundary-dominated flow prevails during the late-time period, and the middle-time pseudo-radial flow period does not exist on the curve. For Case 2, the well is located near the centre of the reservoir and is surrounded by 4 fractures and a hump caused by the decrease in fracture conductivity with production can be found on the curve. In addition, the boundary-dominated flow period is delayed due to the large distance between the well and boundaries.

## Conclusions

Based on our work, the following conclusions can be drawn.

1. A novel fracture model that accounts for the effect of dynamic conductivity as a function of pressure was established. Based on a dimension transformation from a varying-conductivity, equal-length fracture to a constant-conductivity, unequal-length fracture, a new semi-analytical solution for a dynamic-conductivity discrete fracture was developed in the Laplace domain. The new method is simple, computationally stable and accurate.
2. The pressure behaviours of a vertical well on a dynamic-conductivity fracture have been discussed in detail. The effects of boundary size, fracture conductivity and dimensionless permeability modulus on the dimensionless pressure have been presented. The results show that well location mainly affects the early-time flow, dynamic fracture conductivity mainly affects the middle-time flow and large initial fracture conductivity will delay the effect of dynamic conductivity on dimensionless pressure during the middle-time flow period.
3. For a closed rectangular reservoir, the dynamic conductivity of a discrete fracture that depends on the fracture pressure will be reduced dramatically. A small reservoir size will strengthen the effect of dynamic fracture conductivity on dimensionless pressure.
4. The pressure response of a vertical well in the vicinity of a discrete fracture has been revealed. Four flow regimes can be found: early-time radial flow, transitional flow, middle-time pseudo-radial flow and boundary-dominated flow. The middle-time pseudo-radial flow may be masked by the effect of dynamic fracture conductivity. The presence of a fracture near a well is beneficial to production and leads to a reduction in the pressure drop (dip). In contrast, due to decreases in the dynamic conductivity of the fracture with production, a hump can be observed in the middle-time pseudo-radial flow period, which indicates that a steep increase in pressure drop has occurred.
5. In comparison with those for a single fracture near a well, higher production rates can be achieved for wells surrounded by multiple fractures for a given pressure drop. Similar to the single fracture near a well, a hump on the pressure-derivative curve can be found after transitional flow. To take advantage of fractures and weaken the effects of dynamic fracture conductivity in the boundary-dominated flow period, production during early-time radial flow and transitional flow periods becomes more important.

## Electronic supplementary material

Appendix

Supplementary Figures
