# Supplementary material for: Effects of discrete dynamic-conductivity fractures on the transient pressure of a vertical well in a closed rectangular reservoir
Source: Sci Rep. 2017 Nov 14;7:15537. doi: 10.1038/s41598-017-15785-9 (PMC5686130; doi:10.1038/s41598-017-15785-9)
Supplement: Supplementary file 1 — Appendix [file 41598_2017_15785_MOESM1_ESM.doc]

**SUPPLEMENTAL MATERIALS**

**Effects of discrete dynamic-conductivity fractures on the transient pressure of a vertical well in a closed rectangular reservoir**

**Wanjing Luo^1^, Pengcheng Liu^1, †^, Qing Tian^1^, Changfu Tang^2^ & Yinfang Zhou^3^**

**Author Affiliations:**

School of Energy Resources, China University of Geosciences, 29 Xueyuan Road, Beijing, 100083, China.^2^ Exploration Research Institute, Anhui Provincial Bureau of Coal Geology, 20 Najing Road, Hefei, Anhui, 230088, China.^3^ School of Engineering, King's College, University of Aberdeen, Fraser Noble Building, Aberdeen, AB24 3UE, UK.

**^†^Corresponding Authors:**

**Pengcheng Liu**, School of Energy Resources, China University of Geosciences, 29 Xueyuan Road, Beijing, 100083, China. Phone Number: +86 13522168398; Fax Number: +8610-82326850.

E-mail:liupengcheng8883@sohu.com.

**Running Title:**

**The Appendix** of the main article file named as "Effects of discrete dynamic-conductivity fractures on the transient pressure of a vertical well in a closed rectangular reservoir".

**Conflict of Interest:**

The authors declare no competing financial interests.

# Appendix A: Dimensionless definitions of variables

The dimensionless reservoir and fracture pressure

$p_{D}=\frac{kh(p_{i}-p)}{141.2\mu Bq_{w}}$, $p_{fD}=\frac{kh(p_{i}-p_{f})}{141.2\mu Bq_{w}}$ (A-1)

Where: *p_D_* is dimensionless pressure of reservoir, psi; *p* and *p_i_* are respectively pressure and initial formation pressure, psi. *k* is formation permeability, mD; *h* is formation thickness, ft; *µ* is fluid viscosity, cp; *B* is volume factor, RB/STB; *q_w_* is flow rate of a well in the wellhore , STB /d;

The dimensionless time

$t_{D}=\frac{0.0063288k}{\varphi\mu c_{t}x_{f}^{2}}t$ (A-2)

Where: *t* is time variable, d; *t_D_* is dimensionless time; *φ* is porosity, fraction; *c_t_* is the total compressibility, 1/psi; *x_f_* is fracture average half length, ft.

The dimensionless conductivity is

$C_{fD}=\frac{k_{f}\left(p_{fD}\right)w_{f}}{kx_{f}}$ (A-3)

Where: *C_fD_* is dimensionless fracture conductivity changing with fracture pressure, psi; *x_f_* is fracture average half length, ft; *w_f_* is width of the fracture, ft; *p_fD_* is dimensionless fracture pressure; *k_f_* is fracture permeability, mD;

The dimensionless average conductivity is

$C_{fDa}=\frac{1}{N}\sum_{i=1}^{N} C_{fDi}$ (A-4)

Where: *C_fDa_* and *C_fDi_* are the average dimensionless fracture conductivity and dimensionless fracture conductivity of segments "*i*", dimensionless; *N* is the number of segments.

And other dimensionless definitions in the reservoir model

$x_{D}=x/x_{f}$,$y_{D}=y/x_{f}$,$x_{wD}=x_{w}/x_{f}$,$y_{wD}=y_{w}/x_{f}$, (A-5)

$x_{eD}=x_{e}/x_{f}$ $y_{eD}=y_{e}/x_{f}$,$L_{fD}=L_{f}/x_{f}$,$w_{fD}=w_{f}/x{}_{f}$ (A-6)

$x_{f}=\frac{1}{N}\sum_{i=1}^{N} L_{fi}$ (A-7)

The dimensionless wellbore radius is

$r_{wD}=\frac{r_{w}}{x_{f}}$ (A-8)

If the wing has been divided into *N* segments, the total flow rate of the wing is

$q_{fw}=\sum_{j=1}^{N} q_{fj}$ (A-9)

And the dimensionless flow rate of the wing is

$q_{fwD}=\sum_{j=1}^{N} q_{fDj}$ (A-10)

The dimensionless distance between fracture and well is

$D=\frac{d}{x_{f}}$ (A-11)

The dimensionless fracture permeability modulus is

$p_{D}=\frac{141.2\mu Bq_{w}}{kh}\gamma_{f}$ (A-12)

Where: *x* and *y* are respectively *x* and *y* coordinate, ft; *x_e_* and *y_e_* are respectively boundary coordinate in the *x* and *y* direction, ft; *x_D_* and *y*_D_ are respectively dimensionless coordinate in the *x* and *y* direction, ft; *x_wD_* and *y_w_*_D_ are respectively dimensionless wellbore coordinate in the *x* and *y* direction, ft; *L*_f_ is the length of fracture wing; *x*_f_ is the reference length, ft; *L_fD_* and *w_fD_* are respectively dimensionless wing length and width,ft; $\gamma_{f}$is fracture permeability modulus; *D* is dimensionless distance; *d* is distance,ft; *q_fwD_* is dimensionless wellbore flow rate of a fracture; *q_wD_* is dimensionless wellbore flow rate of a well; *r*_w_ is wellbore radial, ft; *r* is radial distance at polar coordinates, ft; *r_wD_* is dimensionless wellbore radial.

# Appendix B: Model of fluid flow in fracture with dynamic conductivity

The flow inside the fracture is assumed to be incompressible and can be described in the 1-Dimensional coordinate. Fluid flow within the fracture obeys Darcy law and the velocity of flow of point *x* can be expressed as

$v(x)=-\frac{k_{f}(p_{f})}{\mu}\frac{\partial p_{f}}{\partial x}$ (B-1)

Multiplying *w_f_ h* in Eq. (B-1), we can obtain the flow rate of point *x,* $q_{c}(x)$

$w_{f}h\frac{k_{f}(p_{f})}{\mu}\frac{\partial p_{f}}{\partial x}+q_{c}(x)=0$ (B-2)

Where: *v*(*x*) is the seepage velocity in the *x* direction, ft/s; *k_f_* is fracture permeability, mD; *p_f_* is fracture pressure, psi; *w_f_* is width of the fracture, ft; *q_c_*(*x*) is the flow rate of point *x,* ft^3^/s; *p_f_* is fracture pressure, psi.

With initial condition

$p_{f}(x,t=0)=p_{i}$ (B-3)

and boundary conditions，

$\left(\frac{\partial p_{f}}{\partial x}\right)_{x=0}=-\left(\frac{q_{c}(x=0)\mu B}{w_{f}k_{f}(p_{f})h}\right)=-\left(\frac{q_{fw}\mu B}{w_{f}k_{f}(p_{f})h}\right)$ (B-4)

$\left(\frac{\partial p_{f}}{\partial x}\right)_{x=x_{f}}=0$ (B-5)

Using the dimensionless definition, we can obtain the following dimensionless equation:

Fluid flow equation,

$C_{fD}(p_{fD})\frac{\partial p_{fD}}{\partial x_{D}}-2\pi\cdot q_{cD}(x_{D})=0$ (B-6)

Eq.(B-6) can also be written as

$\frac{\partial}{\partial x_{D}}\left(C_{fD}(p_{fD})\frac{\partial p_{fD}}{\partial x_{D}}\right)-2\pi\cdot\tilde{q}_{fD}(x_{D})=0$ (B-7)

with initial condition

$p_{fD}(x_{D},t_{D}=0)=0$ (B-8)

and boundary conditions

$\left(\frac{\partial p_{fD}}{\partial x_{D}}\right)_{x_{D}=0}=-\frac{2\pi}{C_{fD}(p_{fD})}q_{fwD}$ (B-9)

$\left(\frac{\partial p_{fD}}{\partial x_{D}}\right)_{x_{D}=1}=0$ (B-10)

Where: *C_fD_* is dimensionless fracture conductivity changing with fracture pressure; $q_{fwD}$ is dimensionless wellbore flow rate of a fracture. *p_fD_* is dimensionless fracture pressure; $q_{cD}$ is dimensionless cross-sectional rate at time t_D_ and point *x*_D_; is dimensionless flow rate strength in the fracture.

Compared with the solution of fracture with uniform conductivity, the solution of dynamic conductivity is more complex because the fracture conductivity *C*_fD_ is a function of *p*_fD_. However, *p*_fD_ is changing with space *x*_D_ and dimensionless time *t*_D_. For a given *t*_D_, we define following transformation,

$\xi_{D}=\xi_{D}(x_{D})=\hat{C}_{fD}(x_{D})\cdot\int_{0}^{x_{D}} \frac{dx_{D}}{C_{fD}(x_{D})}$ , (B-11)

Then the fracture flow Eqs. (B-6)~ (B-10) can be written as,

(B-12)

or

(B-13)

with boundary conditions,

(B-14)

$\left(\frac{\partial p_{fD}}{\partial\xi_{D}}\right)_{\xi_{D}=1}=0$ (B-15)

It is demonstrated that Eqs. (B-12)~ (B-15) has the same form as fracture flow equations for a uniform conductivity fracture with conductivity $\hat{C}_{fD}$.

By integrating Eq. (B-13) twice with the boundary conditions, we can obtain following pressure equation,

(B-16)

In the wellbore,

$p_{fwD}=p_{fD}(0)$ (B-17)

Substituting Eq. (B-17) into Eq. (B-16) and make Laplace transformation, Eq. (B-16) becomes,

(B-18)

Where: $\bar{q}_{fwD}$ is dimensionless flow rate strength in the wellbore in Laplace domain; is dimensionless flow rate strength in the fracture in Laplace domain; $\bar{p}_{fwD}$is bottom pressure *p_fwD_* for a fracture in Laplace domain; *ξ_D_* is dimensionless coordinate in the *ξ* direction; $\hat{C}_{fD}$is uniform conductivity fracture with conductivity.

# Appendix C: Iterative procedure for distribution of conductivity

As stated in Section 2, the dynamic fracture conductivity *C*_fD_ is a function of fracture pressure$p_{fD}$. $p_{fD}$is a function of the location and time. The location variable *x*_D_ can be transformed into variable $\xi_{D}$ with the method discussed in Appendix B. The time variable can be handled in Laplace domain.

For the dynamic-conductivity fracture, we firstly need calculate the distribution of conductivity along the fracture in each time step and then change the varying conductivity model into constant conductivity model by dimension transformation (Appendix B). At last, the classic Cinco-Ley method is used to obtain the solutions.

In each time step, an iterative procedure will be used and following iterative equation in Laplace domain is formulated. Thus, Eq. (B-6) can be written as

$C_{fD}(p_{fD}^{k})\cdot\frac{\partial p_{fD}^{k+1}}{\partial x_{D}}-(2\pi)\cdot q_{cD}^{k+1}=0$ (C-1)

Where: *C_fD_* is dimensionless fracture conductivity changing with fracture pressure; $q_{cD}$is dimensionless cross-sectional rate at time t_D_ and point *x*_D_; *p_fD_* is dimensionless fracture pressure.

The fracture pressure *p*_fD_ calculated from last iterative step *k*-th is used for update the dynamic fracture conductivities. Thus, the transient pressure solution of can be used in each iteration. The iterative procedure is repeated until the wellbore pressure convergence is achieved. The procedures are listed as below.

**(1) Model inputs:** reservoir parameters, fracture parameters, dynamic conductivity parameters.

**(2) Initialization:** *k*=0, set the dynamic conductivity as a constant, i.e. $C_{fD}=C_{fDi}$. The initial pressure$\left(p_{fwD}\right)^{0}$ and $\left(q_{fDi}\right)^{0}$can be obtained by Gaussian elimination method. Then the initial fracture pressure $\left(p_{fDi}\right)^{0}$can be calculated from Eq.( 5) or Eq.(6).

**(3) Iterative procedure:**

Step 1: Calculate the dynamic conductivity $C_{fD}\left(\left(p_{fDi}\right)^{0}\right)$of each segment (Eq.4);

Step 2: Changing the dynamic conductivity into constant conductivity by dimension transformation (Eq.(B-11))

Step 3: Solving equations by Gaussian elimination method and obtaining the parameters$\left(p_{fwD}\right)^{1}$,$\left(q_{fDi}\right)^{1}$; Calculating fracture pressure $\left(p_{fDi}\right)^{1}$ (Eq.(5) or Eq.(6)); Repeating Step 1 and updating the dynamic conductivity $C_{fD}\left(\left(p_{fDi}\right)^{1}\right)$, then repeating Step 2 and Step 3.

……..

Step *K*：If$\left|\left(p_{fwD}\right)^{k+1}-\left(p_{fwD}\right)^{k}\right|<\varepsilon\left(=10^{-6}\right)$then end; otherwise, set $\left(p_{fwD}\right)^{k}=\left(p_{fwD}\right)^{k+1},k=k+1$ and return to step 3 until the convergence is achieved.
