# Supplementary material for: Effects of discrete dynamic-conductivity fractures on the transient pressure of a vertical well in a closed rectangular reservoir
Source: Sci Rep. 2017 Nov 14;7:15537. doi: 10.1038/s41598-017-15785-9 (PMC5686130; doi:10.1038/s41598-017-15785-9)
Supplement: Supplementary file 2 — Supplementary Figures [file 41598_2017_15785_MOESM2_ESM.doc]

**SUPPLEMENTAL MATERIALS**

**Effects of discrete dynamic-conductivity fractures on the transient pressure of a vertical well in a closed rectangular reservoir**

**Wanjing Luo^1^, Pengcheng Liu^1, †^, Qing Tian^1^, Changfu Tang^2^ & Yinfang Zhou^3^**

**Author Affiliations:**

School of Energy Resources, China University of Geosciences, 29 Xueyuan Road, Beijing, 100083, China.^2^ Exploration Research Institute, Anhui Provincial Bureau of Coal Geology, 20 Najing Road, Hefei, Anhui, 230088, China.^3^ School of Engineering, King's College, University of Aberdeen, Fraser Noble Building, Aberdeen, AB24 3UE, UK.

**^†^Corresponding Authors:**

**Pengcheng Liu**, School of Energy Resources, China University of Geosciences, 29 Xueyuan Road, Beijing, 100083, China. Phone Number: +86 13522168398; Fax Number: +8610-82326850.

E-mail:liupengcheng8883@sohu.com.

**Running Title:**

**The Figures** of the main article file named as "Effects of discrete dynamic-conductivity fractures on the transient pressure of a vertical well in a closed rectangular reservoir".

**Conflict of Interest:**

The authors declare no competing financial interests.

**Supplementary Fig. 1:** Wangjing Luo, et al.


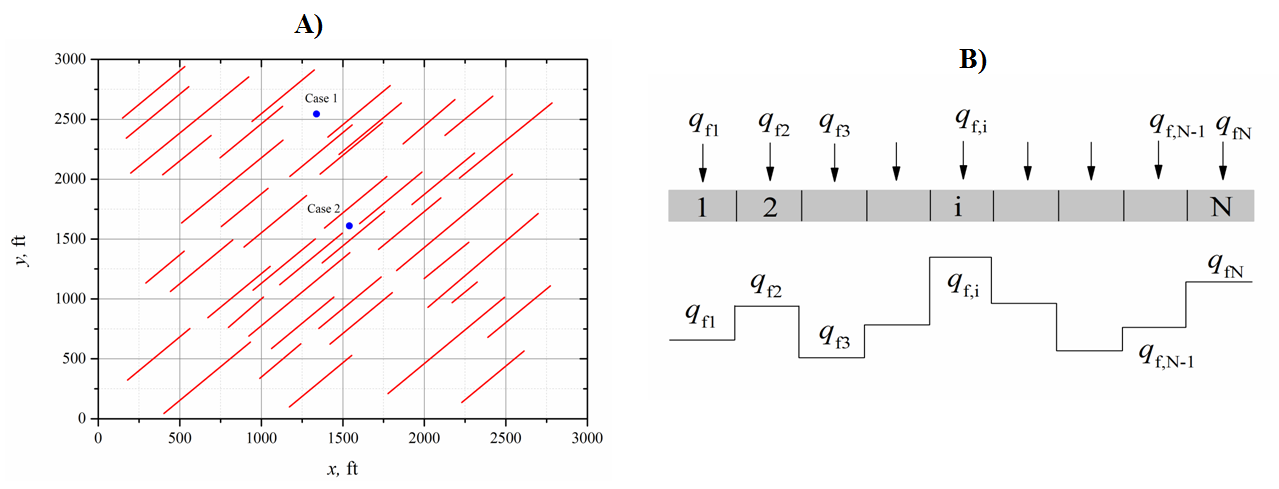


**Supplementary Figure 1.** Schematic of the physical model in a closed rectangular reservoir. **A)** Well with discrete fractures. **B)** Uniform-flux discretization model.

**Supplementary Fig. 2:** Wangjing Luo, et al.


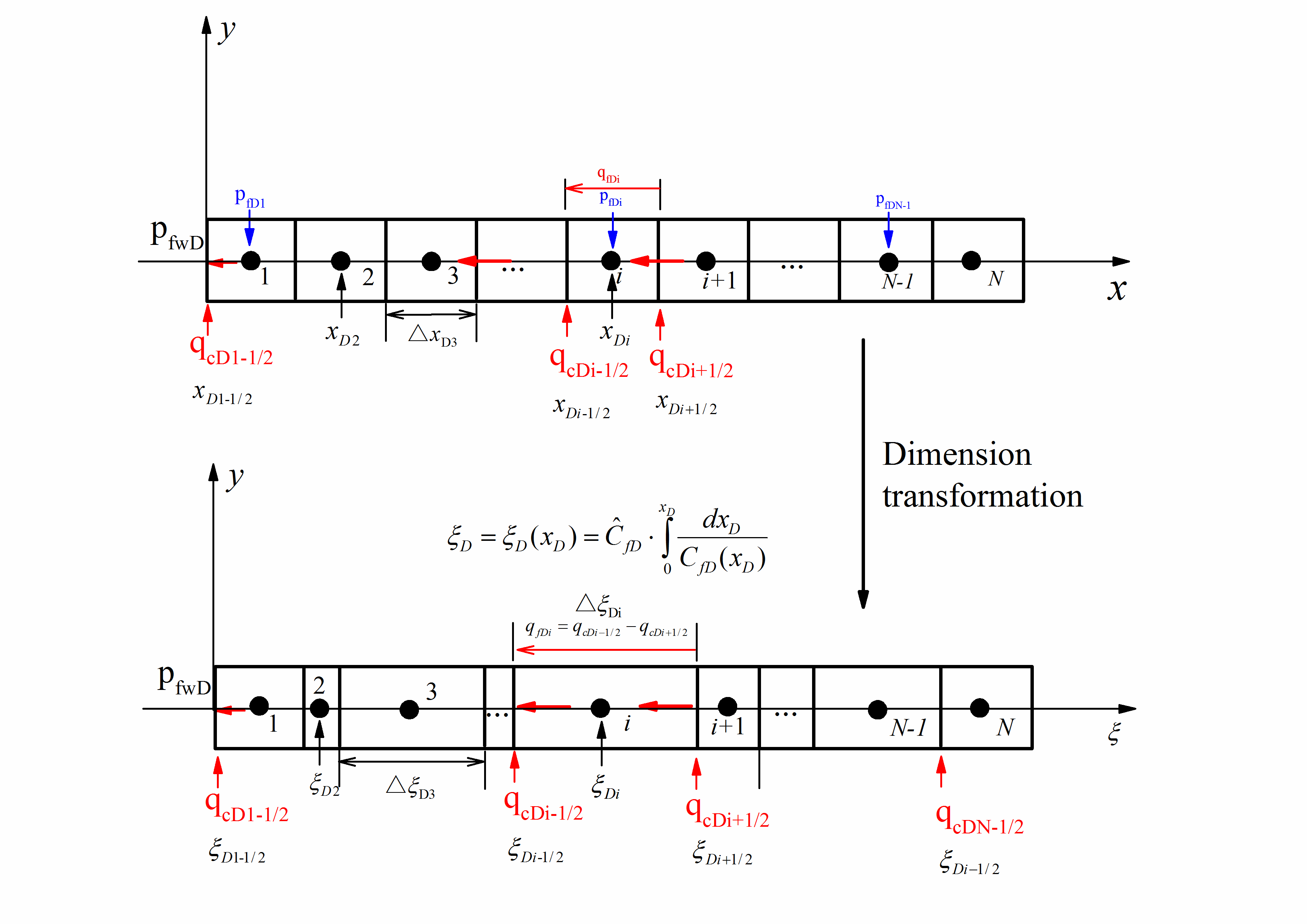


**Supplementary Figure 2.** Schematic of a dimension transformation from a varying-conductivity, equal-length fracture to constant-conductivity, unequal-length fracture.

**Supplementary Fig. 3:** Wangjing Luo, et al.


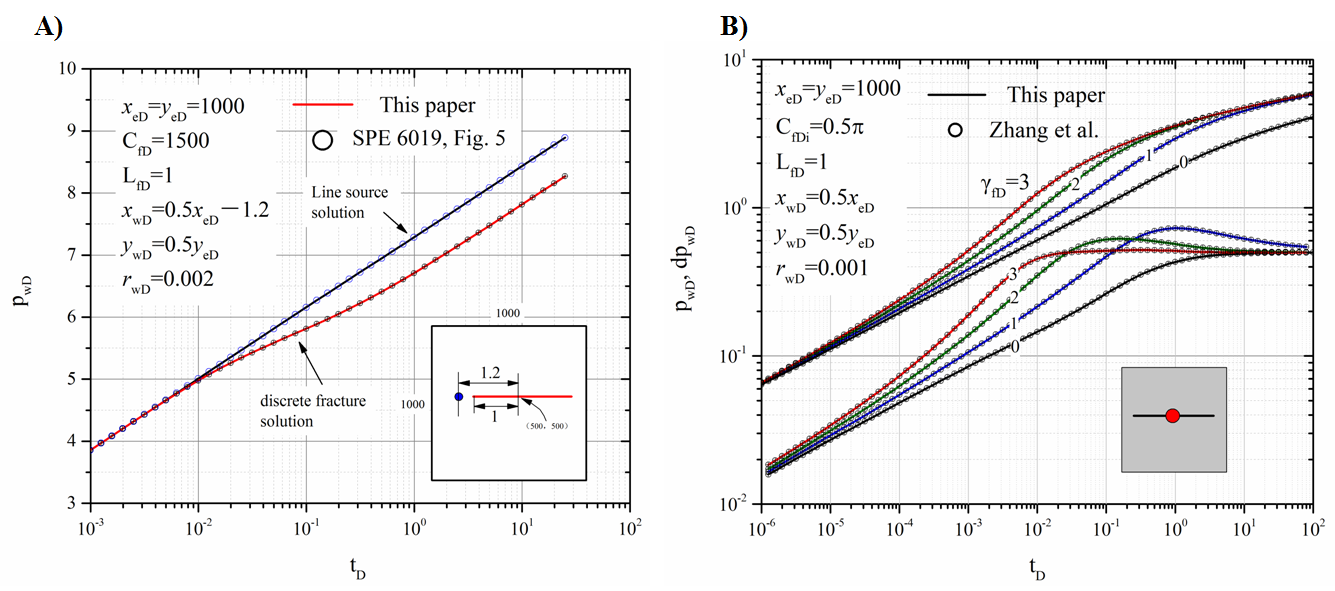


**Supplementary Figure 3.** Comparison of our model with other models. **A)** Cinco-Ley et al. model. **B)** Zhang et al. model.

**Supplementary Fig. 4:** Wangjing Luo, et al.


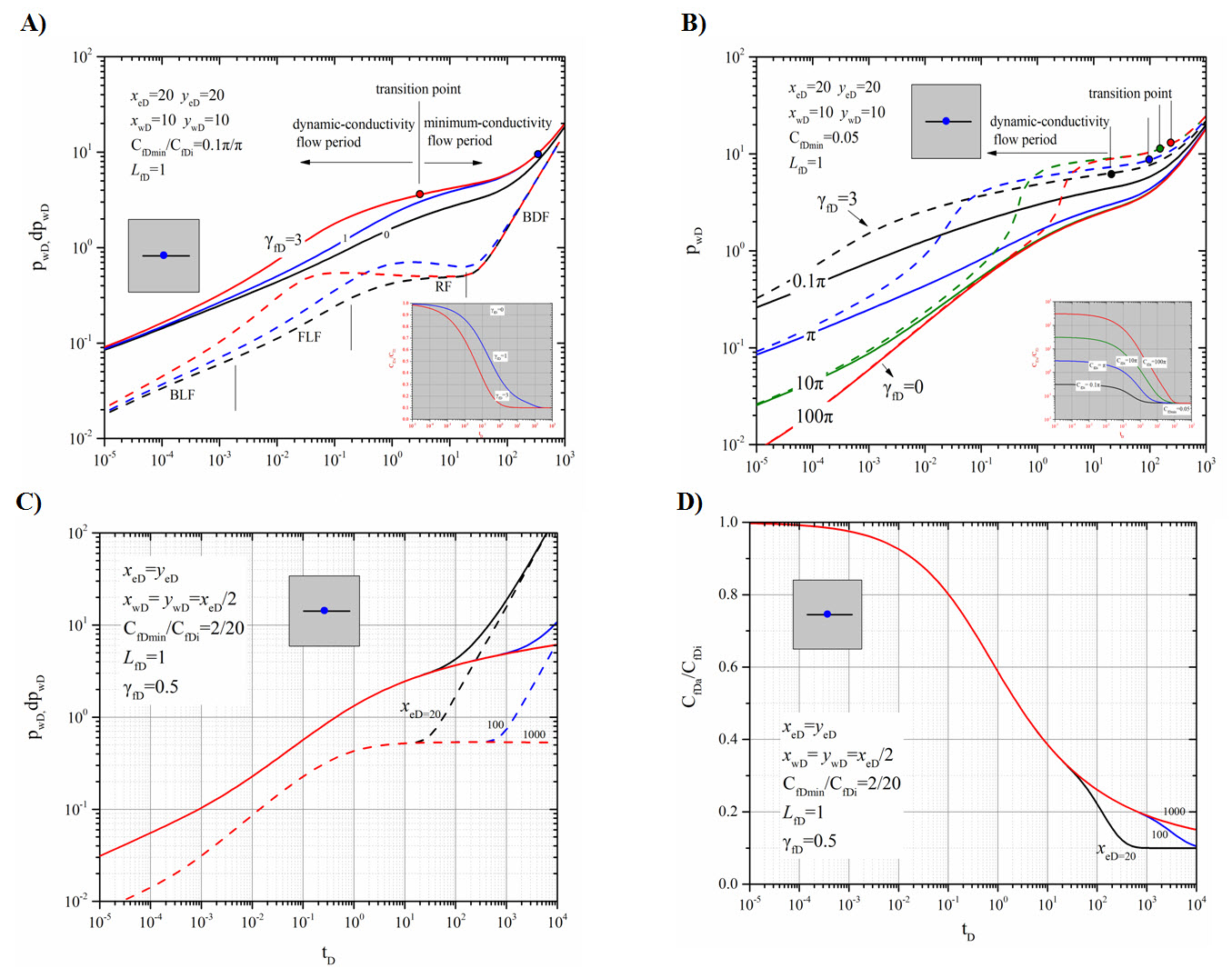


**Supplementary Figure 4.** Effects of fracture permeability modulus and initial dimensionless fracture conductivity on pressure. **A)** Dimensionless pressure (solid lines) and its derivative (dash lines) of a well on a symmetric fracture with different dimensionless fracture permeability moduli. **B)** Dimensionless pressure of a well on a symmetric fracture with different initial dimensionless fracture conductivities. **C)** Effects of boundary size on dimensionless pressure and its derivative on a symmetric fracture. **D)** The ratio of average conductivity to initial conductivity over time for different boundary sizes.

**Supplementary Fig. 5:** Wangjing Luo, et al.


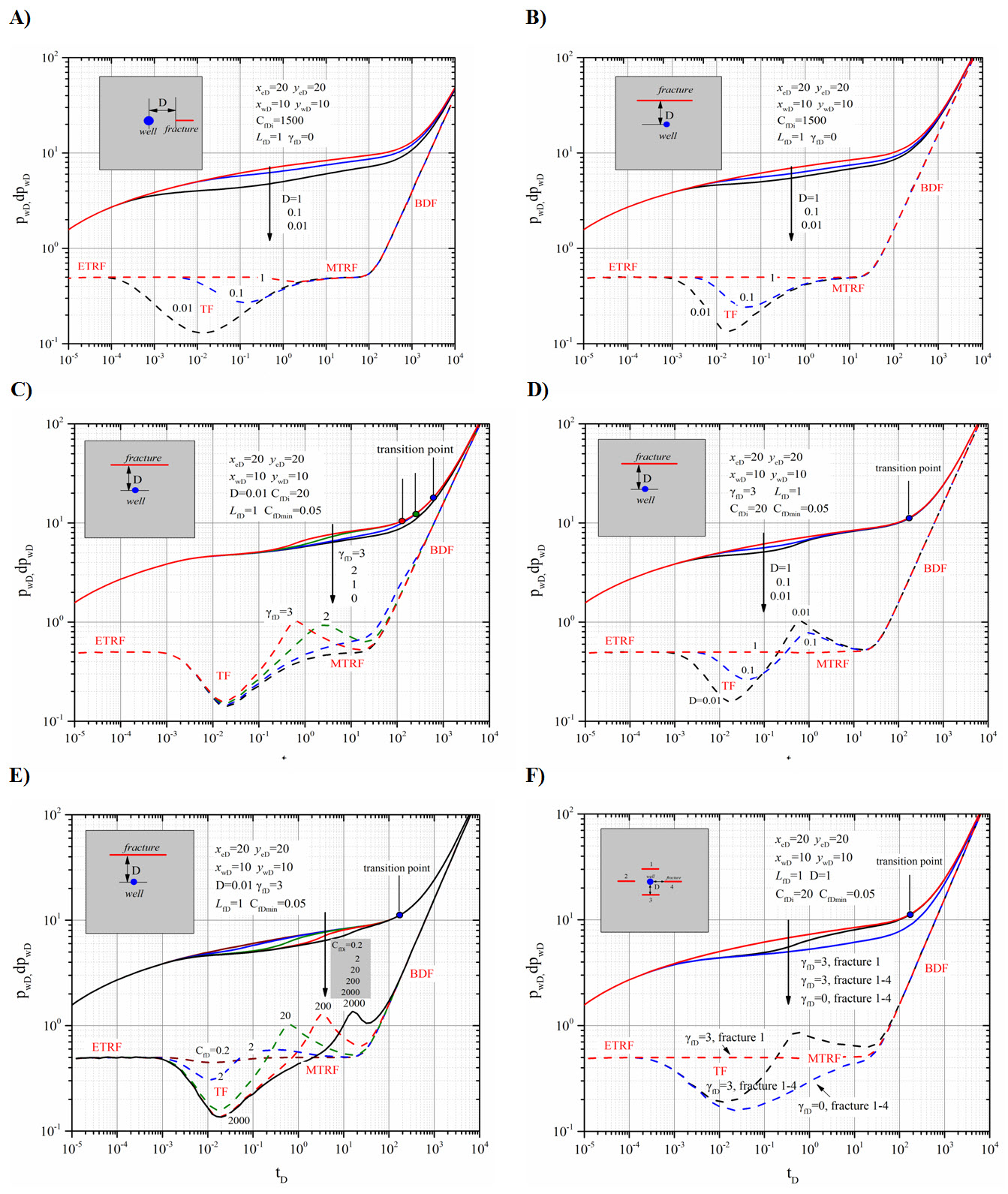


**Supplementary Figure 5.** Effects of parameters on pressure response. **A)** Effects of dimensionless distance on dimensionless pressure and derivative for collinear well and fracture. **B)** Effects of dimensionless distance on dimensionless pressure and derivative for well perpendicular to fracture. **C)** Effects of dimensionless fracture permeability modulus on dimensionless pressure and its derivative. **D)** Effects of dimensionless distance on dimensionless pressure and derivative. **E)** Effects of dimensionless initial fracture conductivity on dimensionless pressure and its derivative. **F)** Comparison of dimensionless pressure and its derivative for a well near a discrete fracture and one surrounded by multiple fractures.

**Supplementary Fig. 6:** Wangjing Luo, et al.


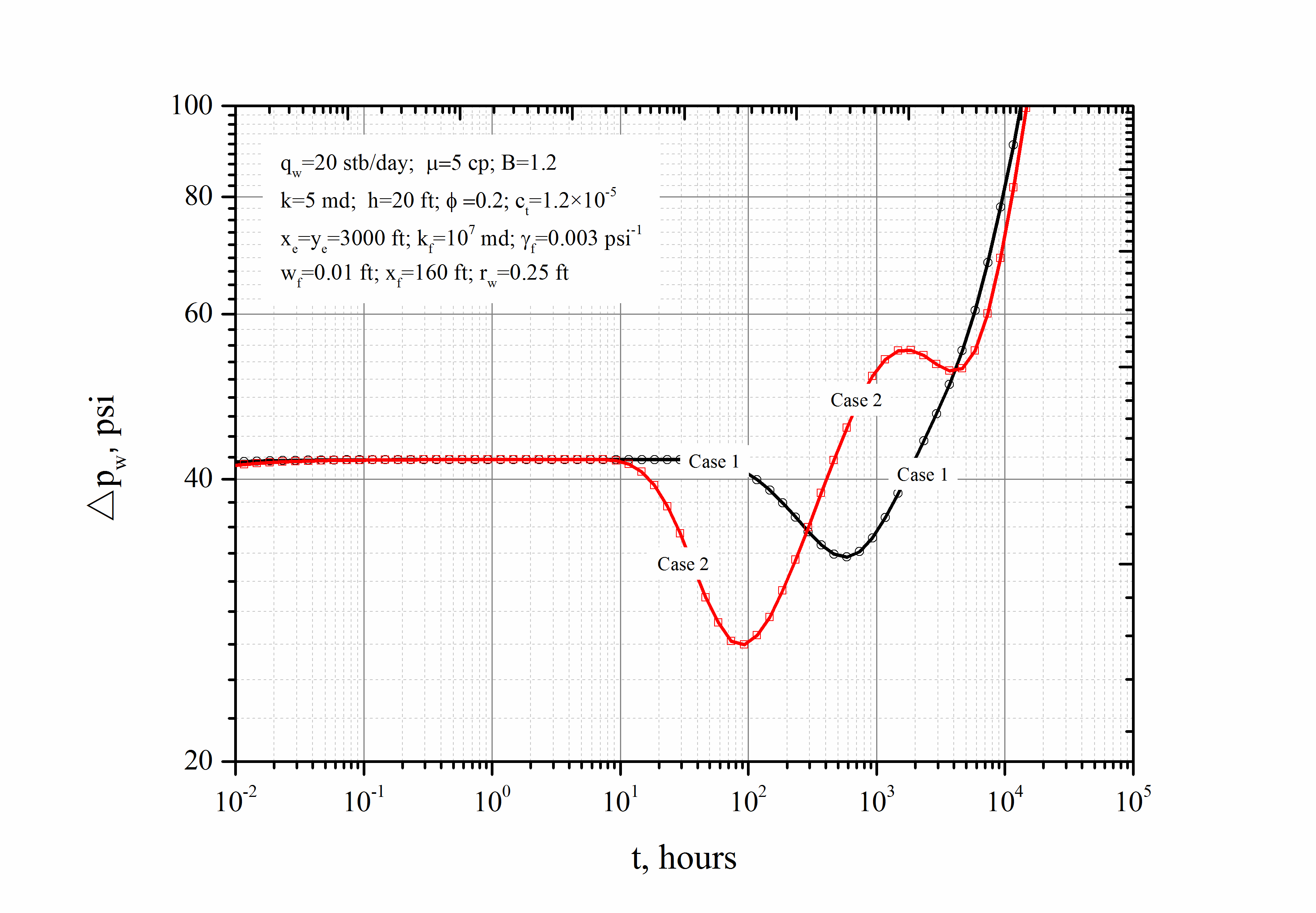


**Supplementary Figure 6.** Pressure derivative in a vertical open hole producing from a formation containing discrete fractures.
